# Investigating the Comparability of Two Multi-Item-Scales for Cyber Bullying Measurement

**DOI:** 10.3390/ijerph15112356

**Published:** 2018-10-25

**Authors:** Julia Fluck

**Affiliations:** Centre for Educational Research, University Koblenz-Landau, 76829 Landau, Germany; fluck@zepf.uni-landau.de; Tel.: +49-6341-280-32179

**Keywords:** cyberbullying, online aggression, measurement

## Abstract

In current cyberbullying literature, researchers assess the phenomenon using a large variety of measurement approaches. This poses a problem in light of comparability of study results. The most common approaches are singular global questions or multi-item scales that list several forms of cyberbullying. Such lists contain either different types of actions or different types of media. This study explores different measurement approaches. Two multi-item scales, one listing different actions and one listing different media, are compared to investigate whether they measure the same latent construct. Confirmatory factor analysis is used to model one factor for each of the multi-item scales. In the first study, the items cover victimization experiences while in the second study they cover estimation of severity. Results show that the two different multi-item scales measure the same latent construct. These results have a substantial impact on the future of cyberbullying research with regard to study comparability.

## 1. Introduction

Cyberbullying is a relatively new phenomenon of aggressive behavior among children and adolescents. Although it has received much attention by the media and the scientific community, many authors criticize that studies on cyberbullying are not based on scientific theories and are all published under the keyword “cyberbullying” in spite of different assessment strategies [1,2]. The assessment of cyberbullying poses many methodological problems (see below). Variations in definitions, operationalizations, and data analysis lead to the problem that results of different studies are hardly comparable.

This special issue focuses on prevention and intervention programs for cyberbullying. Tackling cyberbullying is a very important task since the experience of being bullied online can lead to severe impairments in health and well-being, e.g., psychosomatic problems, anxiety and depression, low self-esteem, and substance abuse [3,4,5]. To evaluate the success of intervention programs, we need reliable and valid measures of the phenomenon. Since different evaluation studies use different operationalizations and measures, we need scientific evidence that different measurement approaches measure the same construct. Thus, measurement is the basis for finding evidence on successful interventions. This paper aims to compare different approaches to ensure valid measurement.

Despite other definitions [2], this paper follows the most common definition by Smith et al. (2008) who understand cyberbullying as “an aggressive, intentional act carried out by a group or individual, using electronic forms of contact, repeatedly and over time against a victim who cannot easily defend him- or herself” [6] (p. 376). Following this definition, studies that ignore the criterion of repetition do not investigate cyberbullying but online (cyber) aggression. “Cyber-aggression refers to any behavior enacted through the use of information and communication technologies that is intended to harm another person(s) that the target person(s) wants to avoid” [7] (p. 305). Therefore, cyberbullying is a subset of online aggression that seems to happen less often.

Beside variations in the defining criteria, which result in different operationalizations, questionnaires vary in other aspects that have been proven to lead to different prevalence rates [8]. In addition to the effect on prevalence rates, those differences in operationalization also lead to diverging correlations with other variables, such as age, gender, and personality [9]. Corcoran and colleagues [10] go as far as stating that different studies on cyberbullying do not even measure the same construct due to differences in operationalization. Those differences concern the following aspects:

- Time frame: The length of the period that subjects are asked to consider has an effect on prevalence. Time frames vary from asking about incidents within the last two months to “ever” [8].
- Cut-off values/frequency: This criterion defines how many attacks a person must experience in order to be considered a victim of cyberbullying. A commonly used criterion is being subject to peer aggression online at least once a week [6]. However, some authors ignore the definition element of repetition and already diagnose cyberbullying when someone has been attacked only once, which is not cyberbullying but online aggression [11]. Mixing up these two phenomena is a big problem in cyberbullying research and leads to artificial effects on prevalence rates of between 4% and 72%, depending on the study [12].
- Choice of wording: Prevalence rates drop when the word “bullying” is explicitly used in an item [13]. This is probably a result of people trying to avoid labeling themselves as a victim [14].
- Measurement strategy: Cyberbullying can be measured directly by asking a singular global question (e.g., “Are you being bullied?”) or by presenting a list of items for different forms of cyberbullying (multi-item scales) [1]. Studies have shown that the two approaches do not lead to the same number of persons identified as bullies or victims, even though correlations between both approaches are high [15,16,17].

For any aspect mentioned above, there is no “right” or “wrong” way of measuring—the adequate strategy depends on the research question. Global questions give information about the subjective perception of a person as a victim, whereas behavior-based multi-item scales try to find out the exact number of incidents.

This paper focuses on multi-item scales and how they relate to each other. A broad variety of multi-item scales can be found in cyberbullying literature [18]. A review of 61 studies found 44 different measurement approaches [19]. Very few studies report data about the reliability or validity of the used instruments so that the quality of measurement cannot be determined [20].

Usually, multi-item scales are based on a specific classification of cyberbullying forms where each form is measured by at least one item. The most commonly used classification approaches in current studies list either different types of actions [21,22] or different types of media [23,24,25]. Taxonomies of media (TOM) distinguish virtual attacks by different types of communication: text messages, e-mails, calls, chats, instant messages, and websites [6]. Taxonomies of actions (TOA) list several types of aggressive behavior in virtual contexts. The taxonomy by Willard (2007) [26] contains the following four forms, which are consistent with the definition of cyberbullying: Harassment is the direct sending of hurtful, offensive, or threatening messages from bully to victim whereas denigration stands for the public humiliation of victims by posting offensive, ridiculing, or other derogatory material. Spreading private messages or pictures/videos that were not meant for the public, against the will of the victim, is called outing/trickery. Exclusion means leaving the victim out of group activities, such as chats, online games, forums, etc.

Taxonomies of actions and taxonomies of media both claim to measure cyberbullying. Studies usually choose one of the two approaches, but none use both multi-item scales. This makes it impossible to compare the results of different studies. Assuming that both courses of action measure the same phenomenon, all studies are listed under the keyword “cyberbullying”. However, this assumption has not been subject to any empirical investigation yet, although there is a strong need to find proof for it [10]. The aim of this study is to find out how multi-item scales based on taxonomies of media and based on taxonomies of actions relate to each other and whether they measure the same latent construct.

## 2. Materials and Methods

In two different empirical studies, teachers in training provided information about bullying and cyberbullying. They informed the students about the definitions of bullying and cyberbullying and about the consequences of victimization, thus pointing out the importance of tackling the problem. After the information session, which was not an intervention, students anonymously filled in questionnaires. As almost all students were under age, the parents received an information form about content and purpose of the study in advance. Their consent was required for the students to participate. If required, the results were communicated to the school administrators in order to get an idea of the extent of cyberbullying problems in their school.

Students from five secondary schools in Germany took part in the first study. *n* = 578 participants filled in the questionnaire. Of these students, 54% were male and 44% were female (2% did not answer the item). The age ranged from 11 to 18 years (*M* = 14.71 years, *SD* = 1.32). *n* = 488 students from three German secondary schools took part in the second study. Of these students, 60.9% were male and 44% were female (one person did not answer the item). The age ranged from 12 to 17 years (*M* = 14.86 years, *SD* = 1.12).

In study 1, the participants filled in a questionnaire that among others contained a global question and two multi-item scales to measure the frequency of experiences with cyberbullying. The scale for the TOA taxonomy contained one item each for harassment (“How often did it occur during the last year that someone sent you threatening, insulting, or other discomforting messages on the internet or on your cell phone?”), denigration (“(...) that someone spread rumors or insults about you throughout the internet or on other peoples’ cell phones?”), outing and trickery (“(...) that someone passed on private e-mails, chat messages, or pictures of you, in order to expose you?”), and exclusion (“(...) that your classmates excluded you from chats or online games?”). The answer format was a five-point Likert scale ranging from “never” to “several times a week.” The scale for the TOM taxonomy used the same answering format and contained items for the following six types of communication: text messages, e-mails, calls, chats, instant messages, and websites (“How often did it occur during the last year that you were attacked, insulted, threatened, exposed, or excluded via the following media…)”.

To answer the research question, a confirmatory factor analysis in Mplus (version 6.1, Muthen & Muthen, Los Angeles, CA, USA) was used to model the latent constructs that the TOA and the TOM are measuring. Models were estimated with the WLSMV algorithm [27] to account for skewed data distributions.

Correlation-based methods such as factor analysis might be susceptible to methodological artifacts. One of the problems with aggression data is low incidence, leading to zero-inflated data, i.e., the majority of people have had no victimization experiences at all, which results in skewed distributions. Another problem is a high poly-victimization across several forms of aggression [28]. Poly-victimization means that people who are victims of one type of aggressive behavior (e.g., violence in schools) also experience other types, such as family violence. A high correlation between two types of aggressive behavior, therefore, cannot prove whether they are both part of the same construct. High correlations between TOA and TOM factors could be due to the fact that many participants hardly experience any victimization at all and those who do, experience different forms of victimization at the same time. To rule out such artifacts, the findings need validation with a second approach.

In study 2, participants also answered questions about several forms of cyberbullying that were listed in one multi-item scale for types of actions and one for types of media. However, the items did not relate to the frequency of experiences but to the severity. Thus, students were asked how “bad” they considered it when someone, e.g., gets a threatening or offensive message (harassment). The answer format was a four-point Likert scale ranging from “not bad at all” to “very bad.” Not only victims of cyberbullying but all students were able to estimate the severity of various bullying forms by putting themselves in the victim’s place, which result in more equally distributed data.

Earlier studies showed that the severity assessment of different cyberbullying forms depend on whether aggressive actions were carried out only by texts or by picture/video material [29]. To consider these differences, harassment, denigration, and outing/trickery were each operationalized by two items, one for text- and one for picture-based virtual attacks.

Confirmatory factor analysis with one TOA and one TOM factor was conducted with Mplus (version 6.1) and the Maximum Likelihood estimator.

To test whether TOA and TOM multi-item scales measure the same latent construct, latent factor correlations were computed for both studies. They can be considered measures of the same latent construct when the latent correlation is very high.

## 3. Results

In Section 3.1 and Section 3.2, the results of study 1 and study 2 are presented.

### 3.1. Study 1

The distribution of answers for victimization experiences with different forms of cyberbullying is skewed with a high inflation of zero experiences (see Table 1).

**Figure 1:**
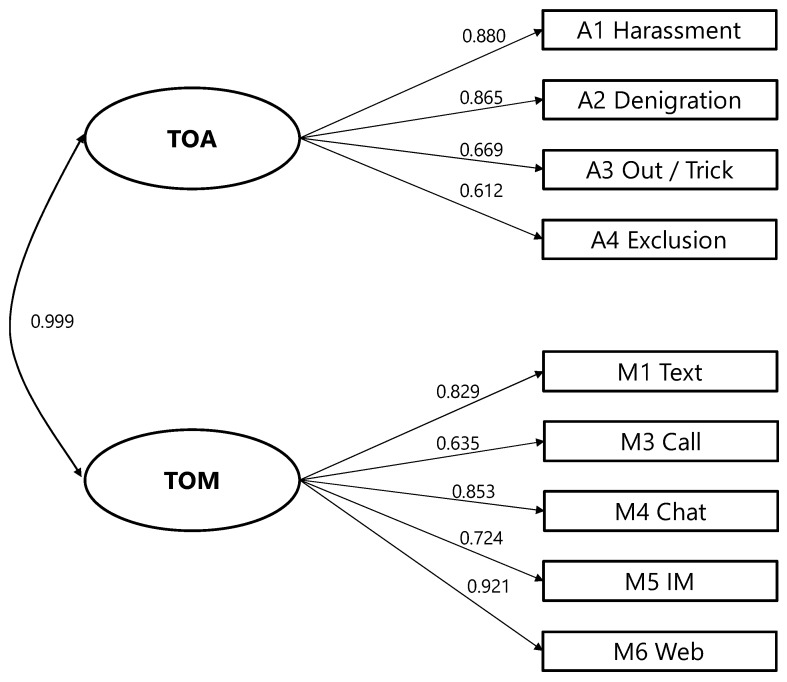
Model 1.1.

Since the item “mail” had a variance close to zero (only 4 of the 558 students ticked off the answer “once or twice”), it was excluded from further analyses. Figure 1 shows the model, factor loadings, and factor correlations of the confirmatory factor analysis with two separate factors (model 1.1).

All factor loadings are significant (*p* < 0.001) and show a large connection to the factors (0.612 < λ < 0.921). The latent correlation between TOA and TOM is so high that it is evidently not different from a perfect correlation. This can be tested by comparing the specified model to a second model (model 1.2) that consists of only one factor; see Table 2. Mathematically, this is equal to fixing the correlation at 1. A chi² difference test shows no significant difference in model fit. The chi² difference test for nested models investigates whether model fit decreases significantly when parameters are fixed that were free in a H0 model. Since a direct comparison with the baseline model is not available for WLSMV, this was done with the DIFFTEST function in Mplus. The measures for global fit show that the models do not significantly differ from each other and fit the data well (Table 2). The two factors for TOA and TOM measure the same latent construct.

### 3.2. Study 2

Table 3 shows the descriptive statistics and the distribution of answers for the estimated severity of different forms of cyberbullying. Picture-based forms are in each case considered more severe than the respective text-based forms. The items in the TOA multi-item scale show a wider range in severity than the TOM items.

**Figure 2:**
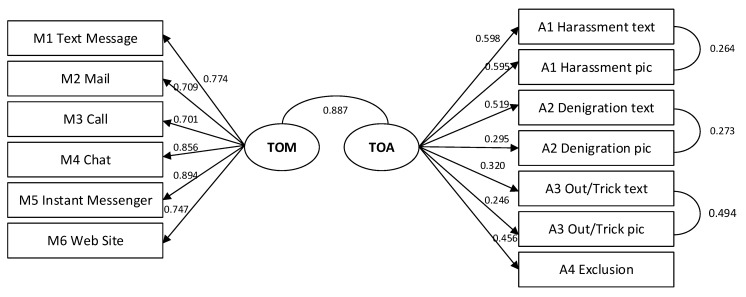
Model 2.1.

Model 2.1 replicates the two-factor model in study 1. Since text- and picture-based forms of each type of action share more variance than they do with other forms of cyberbullying, additional correlations are specified. Figure 2 shows the model and its estimation results.

All factor loadings in model 2.1 are significant (*p* < 0.001) and show a substantial connection to the factors (0.246 < λ < 0.894), although the factor loadings are lower than in study 1. The latent correlation between the two factors is very high but as a chi² difference test with a one-factor model shows, the correlation is significantly different from one (see Table 4). As a result, factor loadings on a common factor are still significant but somewhat lower (0.217 < λ < 0.889) and the model fit is better in a two-factor model.

For both models, model fit is acceptable. Evidently, the very high factor correlation still speaks for a common underlying construct.

## 4. Discussion

The frequency of victimization approach in study 1 and the severity approach in study 2 complement each other and give insight in two different perspectives of cyberbullying measurement with multi-item scales.

The results indicate that TOA and TOM measures are both indicators of the same latent construct. As expected, data from the severity approach, which are not susceptible to artifacts due to poly-victimization and zero-inflated data, show somewhat lower factor loadings and correlations than data on the frequency of experience. However, the second study supports the assumption of a common construct behind both measurement approaches.

This finding is very important for the future of cyberbullying research. Like many authors have complained [2,10], different studies on cyber aggression and cyberbullying are hardly comparable because of the different definitions, operationalization, etc. that they use. The results of this study show, however, that studies with multi-item scales by TOA or by TOM can be compared in spite of their different operationalization.

For correlation studies with other constructs, as well as for the evaluation of prevention and intervention programs, researchers have to decide on an adequate measurement strategy. Using TOA as well as TOM multi-item scales is the best approach because more items lead to a more reliable and, therefore, a more valid measurement. Due to economic reasons, it might become necessary to restrict oneself to the use of only one of the two multi-item scales. Although TOA taxonomies give a more detailed picture of what exactly the cyberbullying incidents look like, TOM scales are probably the better choice for intervention studies because they show which media represent the highest risks. TOA scales should differentiate between text- and picture-based forms of attacks. When the researcher is less interested in the number of attacks and more in the subjective perception of the victims, singular global measures can complement multi-item scales with an inside perspective.

There are also a few limitations to consider. While this paper shows that results of different studies can be compared when they use either the taxonomy of actions [6] or the taxonomy of media [26], some studies on cyberbullying use neither of the two measures. For such different approaches, the proof of equivalence is still needed. The procedure described in this paper can be a blueprint for testing the equivalence of other measurement approaches. In addition, since the data were collected from ad-hoc samples, the results should be replicated with larger and representative samples.

## 5. Conclusions

“Cyberbullying research has addressed substantive problems and between-construct issues before within-construct issues such as definition, structure, and measurement have been resolved” [30] (p. 68). This paper dealt with an important within-construct issue, the measurement of cyberbullying. As the results show, data from existing studies can be compared even though they use different approaches to assess cyberbullying. At the same time, the same procedure can be applied to other operationalizations of cyberbullying, thus finding out whether even more measurement approaches lead to comparable results. The fact that the issue of measurement is often ignored in cyberbullying literature is problematic since measurement is the basis for assessing the success of prevention and intervention. Without reliable and valid measurement, however, it is impossible to identify best-practice intervention programs.

This article focuses on measurement, but important information was also gained about another unresolved issue on the concept of cyberbullying, concerning its structure [19,30]. It seems that in spite of different types of actions and types of media, which are used to cyberbully others, the construct is heterogeneous yet unidimensional.

Early interventions to tackle cyberbullying are becoming more and more important because of the consequences of cyberbullying experiences. Lately, adults—even in visible political and celebrity positions—have been setting a bad example by openly engaging in behaviors of online social cruelty. This can lead to an acceptance of such misbehaviors among adolescents and makes effective interventions difficult, but all the more important.

However, although the internet seems to be a dangerous place for children and adolescents where they are at risk of experiencing bullying, sexual harassment or witnessing excessive violence [31], the web and information and communication technologies can also be used as tools for intervention [32]. As the research field of homophobic and transphobic bullying shows, for example, risks of anonymous online harassment are balanced by the benefits of online connections and online activism for marginalized youth [33].

## Figures and Tables

**Table 1:** Descriptive statistics and distributions for both multi-item-scales in study 1.

Item		*M*	*SD*	Number of Answers per Category ^1^				
				(1)	(2)	(3)	(4)	(5)
Type of action								
A1	Harassment	1.34	0.701	417	109	17	7	6
A2	Denigration	1.34	0.744	423	99	22	3	10
A3	Out/Trick	1.11	0.417	507	39	3	4	1
A4	Exclusion	1.10	0.441	518	27	5	2	3
Type of medium								
M1	Text	1.19	0.495	473	74	8	2	2
M2	Mail	1.01	0.084	554	4	—	—	—
M3	Call	1.14	0.530	507	36	6	3	5
M4	Chat	1.22	0.591	458	71	12	4	4
M5	IM	1.10	0.445	517	31	—	4	3
M6	Web	1.33	0.737	426	99	14	9	8

^1^ Answers range from (1) = “no experiences at all” to (5) = “several times a week”.

**Table 2:** Model fit and model comparison for two- (1.1) and one-factor (1.2) models in study 1.

Fit Index	Model 1.1	Model 1.2
chi² (df), *p*	75.538 (26), *p* < 0.00001	75.512 (27), *p* < 0.00001
RMSEA (c. i.)	0.060	0.058
CFI	0.988	0.988
TLI	0.983	0.984
chi² difference test	1.001 (1), *p* = 0.3171	

**Table 3:** Descriptive statistics and distributions for both multi-item scales in study 2.

Item		*M*	*SD*	Number of Answers per Category ^1^			
				(1)	(2)	(3)	(4)
	Type of action						
A1T	Harassment text	2.67	0.809	32	165	209	71
A1B	Harassment picture	3.20	0.753	13	58	227	180
A2T	Denigration text	3.37	0.772	12	50	163	253
A2B	Denigration picture	3.59	0.727	12	32	95	337
A3T	Outing/Trickery text	3.44	0.737	7	50	149	273
A3B	Outing/Trickery picture	3.72	0.602	6	20	76	374
A4	Exclusion	2.19	0.892	118	183	141	34
	Type of medium						
M1	Text	2.65	0.809	37	158	217	64
M2	E-Mail	2.52	0.880	67	149	201	57
M3	Phone call	3.02	0.909	38	79	198	163
M4	Chat	2.65	0.854	44	152	204	75
M5	Instant Messenger	2.68	0.870	46	141	205	81
M6	Website	2.97	0.835	20	113	205	140

^1^ Answers range from (1) = “not bad at all” to (4) = “very bad”.

**Table 4:** Model fit and model comparison for two- (2.1) and one-factor (2.2) models in study 2.

Fit Index	Model 2.1	Model 2.2
chi² (df), *p*	185.286 (61), *p* < 0.00001	197.547 (62), *p* < 0.00001
RMSEA (c. i.)	0.067	0.069
CFI	0.950	0.945
TLI	0.936	0.931
chi² difference test	12.261 (1), *p* = 0.0005	
